# Photodynamic Therapy for Eye Cancer

**DOI:** 10.3390/biomedicines5040069

**Published:** 2017-12-08

**Authors:** Paul Rundle

**Affiliations:** Ocular Oncology Service, Royal Hallamshire Hospital, Sheffield S10 2JF, UK; paul.rundle@sth.nhs.uk

**Keywords:** photodynamic therapy, photodynamic therapy, uvea, melanoma, metastasis, choroid, eye, cancer

## Abstract

Photodynamic therapy is well-established as a treatment for a number of conditions in ophthalmology, principally in the field of medical retina, but less so in ocular oncology. Cancer of the eye is rare, the commonest lesions to affect the globe being choroidal melanoma (as a primary malignancy) and choroidal metastases (a secondary malignancy). The mainstay of treatment of such lesions remains radiotherapy in various forms, however, photodynamic therapy does have a useful role to play in the management of such patients. In this article, I hope to review the current indications, treatment regimes, and the risks and benefits of photodynamic therapy (PDT) as a treatment for eye cancer.

## 1. Introduction

Cancer affecting the intraocular compartment of the eye is rare. The commonest lesions to do so are primary melanoma of the uveal tract (iris, ciliary body and choroid) and metastatic (secondary) tumours to the uveal tract from cancers elsewhere. These two conditions are quite distinct and the management options differ depending on the diagnosis.

Photodynamic therapy (PDT) using verteporfin as the photoactive agent is a well-established treatment for a number of conditions affecting the retina [1] however the use of PDT in treating ocular cancer is less well-established. In this article I will review the current evidence for its use as well describing my own experience of treating more than 100 such patients.

## 2. Choroidal Melanoma

Melanoma affecting the uveal tract is the commonest primary intraocular malignancy in adults, affecting approximately 5–9 per million population per year [2,3]. It principally affects Caucasian individuals and is extremely uncommon in non-white races [3]. The aetiology is unclear however, despite this racial variation, unlike with skin melanoma; there is no evidence of a link between UV exposure and choroidal melanoma. The condition most commonly affects adults in the 50–80 years age range, with no significant difference between the sexes and is almost invariably unilateral [4]. Approximately 50% of individuals diagnosed with uveal melanoma will die of metastatic disease (principally to the liver) [5]. The risk of metastasis correlates with certain chromosomal abnormalities within the tumour principally monosomy 3 and multiple copies of chromosome 8 [6]. Unfortunately, response rates to current treatments for metastatic uveal melanoma remain low.

A number of treatment options exist for uveal melanoma including plaque brachytherapy using a variety of isotopes (principally ruthenium-106 [7] and iodine-125 [8]), proton beam therapy [9], stereotactic radiosurgery [10] and eye removal [8] (enucleation or exenteration in advanced cases with orbital extension). A variety of laser techniques such as argon laser photocoagulation [11] and diode laser transpupillary thermotherapy (TTT) have been used in the past but have largely been abandoned because of the risk of late recurrence [12]. Photodynamic therapy (PDT) utilizing verteporfin as the photoactive agent is not a widely-used treatment for uveal melanoma. This may be partly due to the fact that some of the earliest case reports of the use of PDT described its use in treating tumours that had not responded to other treatments such as radiotherapy or diode laser thermotherapy suggesting that these lesions may have been particularly resistant tumours [13]. Secondly, these initial reports often described the use of a single session of PDT treatment which may have been insufficient particularly as later case series involving multiple sessions of treatment have shown more promising results [14,15,16,17].

In general in ocular oncology the treatment regime used for PDT is the TAP (Treatment of Age-related macular degeneration with PDT) protocol [18] or a variant thereof. The TAP protocol involves a dose of 6 mg/m^2^ verteporfin given by intravenous infusion over a 10-min period. Then, 5 min following completion of the infusion, the target tissue is illuminated with coherent light of 690 nm wavelength at an intensity of 600 mW/cm^2^ for 83 s per application. This results in 50 J/cm^2^ of delivered energy. In this setting, verteporfin PDT has been shown to be safe with a low incidence of systemic side effects. Significant, albeit rare, reactions include infusion-related back pain (2.2%) and pruritus (2%) [19]. One advantage of the use of verteporfin is the relatively short period of post-treatment photosensitivity lasting approximately 48 h. Although the total energy delivered varies between studies the consensus seems to be to use a fluence of 100 J/cm^2^ involving multiple applications to cover the surface of the melanoma and adjacent tissues [15,16,17]. The results of the three largest studies by Rundle [15], Fabian [17] and Campbell [16] are summarized in Table 1 and Table 2.

Although the study populations are not identical, combining the results of these three studies totaling 42 patients showed a local control rate of between 80% and 89%. This compares to up to 95–97% local control for proton beam radiotherapy [9] and stereotactic radiosurgery [20]. The difference however is that with PDT, vision is often maintained, if not actually improved with PDT with only two out of 42 patients in the studies in Table 1 showing a loss of vision of more than two lines of Snellen acuity. In contrast, patients undergoing proton beam or stereotactic radiosurgery may expect significant visual loss of >3 lines of Snellen acuity in 45% to 65% of cases [20]. Furthermore, the risk of secondary enucleation is higher with such radiotherapy amounting to 3.7–14% for proton beam therapy [20,21] and 2.4–14% for stereotactic radiosurgery [20,22]. There have been no reports of secondary enucleation with PDT thus far using the multidose treatment described in the papers in Table 1. Other advantages of PDT is that it is performed as an outpatient requiring no general or regional anaesthesia, it may be repeated as required and the costs are small compared to those of proton beam or stereotactic radiosurgery.

**Figure 1:**
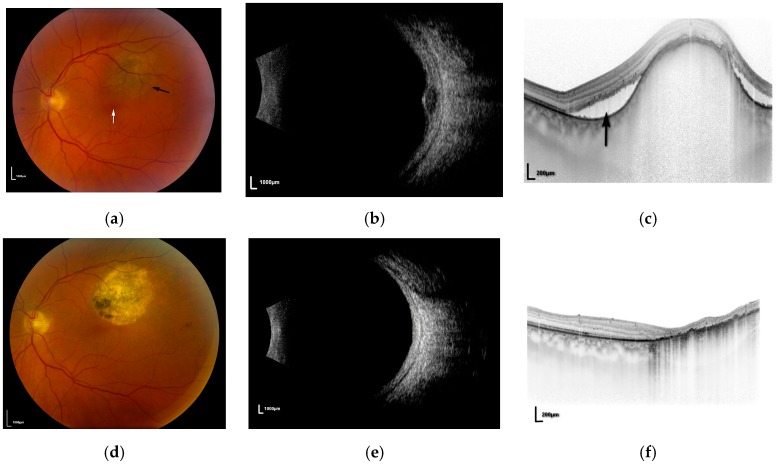
(**a**) Fundal photograph of left eye showing melanoma (**black arrow**) adjacent to the fovea (**white arrow**); (**b**) ultrasound of lesion in Figure 1a; (**c**) ocular coherence tomography (OCT) scan demonstrating sub-retinal fluid (**black arrow**); (**d**) post-treatment fundal photograph showing flat scar; (**e**) post-treatment ultrasound demonstrating flat scar; (**f**) post-treatment OCT showing resolution of sub-retinal fluid.

The question remains, which patients with uveal melanoma are suitable for PDT? It appears that amelanotic (non-pigmented) tumours fare better than pigmented lesions although the assessment of the degree of pigmentation is subjective. In the author’s experience of treating almost 80 patients (unpublished data) the ideal tumour is pale with a significant amount of associated sub-retinal fluid. This may relate to the degree of vascularity within these “leaky” tumours. The patient shown in Figure 1 presented with an amelanotic melanoma (Figure 1a), measuring slightly less than 2 mm in thickness (Figure 1b) situated less than 1 mm from the fovea. Snellen visual acuity was reduced to 6/12 owing to sub-retinal fluid extending beneath the fovea, demonstrated on ocular coherence tomography (OCT) scanning (Figure 1c). Following three sessions of PDT the tumour regressed to a flat scar (Figure 1d,e) with resolution of all subretinal fluid (Figure 1f) and her vision returned to 6/5. There has been no recurrence at 2 years follow up.

It has been noted previously that a small proportion of patients receiving PDT for amelanotic tumours develop severe pain 24–48 h post-operatively, requiring a short course of systemic steroids [15,16]. These tumours tend to show a marked regression following the first session of PDT suggesting that pain may be due to scleritis relating to brisk tumour necrosis [15].

Tumours that are pigmented, particularly those with a flat pigmented edge seem to respond less well to PDT [17]. The reason for this is unclear however it may be that significant amounts of melanin pigment within the tumour hinders absorption of the laser energy. Furthermore, the flat peripheral margins of pigmented tumours may be relatively avascular compared to the thicker central portions so limiting the accumulation of the photosensitizing agent within these flatter areas. None of the patients with pigmented tumours in the study by Fabian [17] suffered post-operative pain or scleritis which may reflect the lesser response to PDT in these cases.

## 3. Choroidal Metastases

Choroidal metastases (secondary tumours to the eye) are the commonest type of cancer to affect the adult eye with post-mortem studies suggesting that 8% of individuals dying of cancer show histological evidence of metastases within the eye [23]. That being said, in clinical practice, only a minority of such patients present to ocular oncologists and this may be because they are asymptomatic or the problem remains undetected in the context of more widespread and often pre-terminal metastatic disease.

Owing to the vascularity of this layer, the choroid is the commonest site for metastases within the eye [24] and in theory any tumour spreading by the haematogenous route could metastasize to the eye. That being said a number of cancers show a predilection for the eye with the commonest tumours being breast and lung carcinoma [24]. In a significant number of cases the source of the metastases is never found (cancer of unknown primary) [24]. In cases of breast carcinoma, metastasis to the choroid may occur many years (>10) after the original primary even in the absence of any other obvious recurrence or metastatic disease. In contradistinction, choroidal metastases from some cancers notably lung carcinoma may be the first sign of cancer in the patient [24].

There are a number of treatment options for choroidal metastatic disease including radiotherapy (external beam [25] or brachytherapy [26]), systemic chemotherapy [27] and photodynamic therapy [28]. Which option is preferred depends on a number of factors including location and extent of the metastases, whether one or both eyes are affected and the sensitivity of the primary cancer to chemotherapy. More recently intraocular injections of anti-vascular endothelial growth factor (VEGF) agents have been tried with varying degrees of success [29,30].

**Figure 2:**
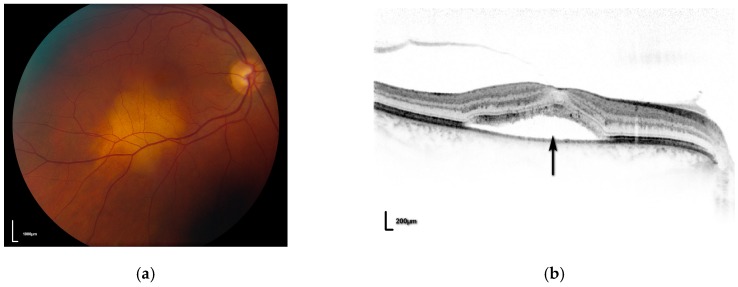

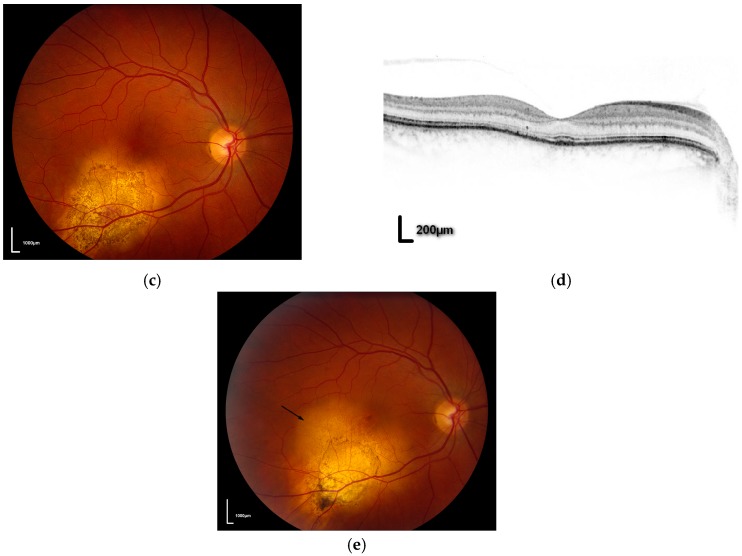
(**a**) pre-treatment fundal photograph of right eye demonstrating metastasis; (**b**) OCT scan demonstrating fluid beneath the fovea (**black arrow**); (**c**) post-PDT photograph showing regression of metastasis; (**d**) post-PDT OCT scan showing resolution of fluid; (**e**) fundal photograph showing recurrent tumour at edge of treatment scar (**black arrow**).

A number of small case series have described the successful use of photodynamic therapy as a treatment for choroidal metastatic disease [28,31]. The protocol used tends to be the TAP protocol, using a spot duration of 83 s to deliver 50 J/cm^2^ rather than the 100 J/cm^2^ used for uveal melanoma. The advantages of PDT for such patients are that treatment is given as a day procedure, requiring only topical anaesthesia and may be repeated as required. In the largest series to date Ghodsara [28] treated a total of 21 metastatic deposits in 13 eyes of 10 patients with regression of 76% (16 tumours), stable disease in 10% (2 tumours) and continued growth in 14% (3 tumours). Visual acuity improved in four eyes, remained stable in three eyes and decreased in six eyes. Kaliki et al. [31] treated nine tumours in eight eyes of eight patients with regression of seven tumours (78%) and continued growth of two lesions (22%). In their series visual acuity was stable or improved in seven eyes (87.5%) and deteriorated in one eye (12.5%). This compares favorably with results from fractionated external beam radiotherapy (EBRT). Wiegel et al. [25] reported visual stability or improvement in 86% of patients receiving EBRT however it must be remembered that such treatment may require anything up to 15–20 daily sessions of treatment which may be difficult for patients suffering from potentially terminal metastatic disease. It is interesting to note that none of the patients reported developed significant pain or scleritis following PDT. The present author’s experience of PDT for choroidal metastases is similar to the published reports mentioned above (unpublished data) with visual results depending on the anatomical location of the tumour. The patient in Figure 2 presented with a choroidal metastasis (arising from a primary breast adenocarcinoma) in the right eye (Figure 2a) with fluid extending beneath the fovea (Figure 2b). Following one session of PDT the tumour regressed (Figure 2c) with resolution of sub-retinal fluid (Figure 2d) and visual acuity returned to 6/9. In the author’s experience, metastatic deposits frequently show marked central regression/necrosis post-PDT, but less of a response towards the thinner periphery of the tumour. The peripheral portion of such tumours tends to be less vascular than the thicker central portion and it may be this relative avascularity of the periphery that allows for late tumour recurrence in these patients (Figure 2e).

## 4. Conclusions

Although photodynamic therapy is well-established in ophthalmic practice, its use remains controversial in the field of ocular oncology, largely because of the lack of large case series with long-term follow-up. Nevertheless, with appropriate case selection, PDT appears to be a safe and well-tolerated treatment with excellent local control rates and a good visual prognosis for many patients.

## Figures and Tables

**Table 1:** Tumour characteristics and treatment parameters.

Reference	Number Treated	Pigmented (%)	Thickness Range/mm	Fluence J/cm^2^	Follow-Up/Months
[15]	18	9 (50%)	0.5–4.4	100	10–42
[17]	15	15 (100%)	0.9–2.7	100	8–18
[16]	9	0	1.3–5.7	50 (50%) 100 (50%)	34–90

**Table 2:** Local control rates and visual results.

Reference	Regressed (%)	Recurrence/Failure (%)	Vision Improved (%)	Vision Stable (%)	Visual Impairment (%)
[15]	16 (88)	2 (12)	6 (33)	10 (56)	2 (11)
[17]	12 (80)	3 (20)	Not stated	Not stated	0
[16]	8 (89)	1 (11)	3 (33)	6 (67)	0

Visual impairment defined as loss of >2 lines of Snellen acuity.
